# Computer-aided identification of *Mycobacterium tuberculosis* resuscitation-promoting factor B (RpfB) inhibitors from *Gymnema sylvestre* natural products

**DOI:** 10.3389/fphar.2023.1325227

**Published:** 2023-11-29

**Authors:** Mohibullah Shah, Fatiha Khan, Iqra Ahmad, Cun-Liang Deng, Asia Perveen, Anwar Iqbal, Umar Nishan, Aqal Zaman, Riaz Ullah, Essam A. Ali, Ke Chen

**Affiliations:** ^1^ Department of Biochemistry, Bahauddin Zakariya University, Multan, Pakistan; ^2^ Department of Infectious Diseases, The Affiliated Hospital of Southwest Medical University, Luzhou, China; ^3^ Department of Chemical Sciences, University of Lakki Marwat, Lakki Marwat, Pakistan; ^4^ Department of Chemistry, Kohat University of Science and Technology, Kohat, Pakistan; ^5^ Department of Microbiology and Molecular Genetics, Bahauddin Zakariya University, Multan, Pakistan; ^6^ Department of Pharmacognosy, College of Pharmacy, King Saud University, Riyadh, Saudi Arabia; ^7^ Department of Pharmaceutical Chemistry, College of Pharmacy, King Saud University, Riyadh, Saudi Arabia

**Keywords:** multi-drug resistance, tuberculosis, RpfB, dormancy, *Gymnema sylvestre*, *in-silico*

## Abstract

Tuberculosis (TB), an infectious disease caused by multi-drug resistant *Mycobacterium tuberculosis* (Mtb), has been a global health concern. Mtb affects over a third of the world’s population, causing two million deaths annually due to its dormancy and propensity to spread infection during this period. Resuscitation-promoting factor B (RpfB) plays a pivotal role in the growth of Mtb during dormant periods, making it a critical target for eliminating Mtb and curing TB. Gymnema sylvestre is a famous medicinal plant with several medicinal properties, including antimicrobial activity; however, the therapeutic potential of the various reported metabolites of this plant against Mtb has not yet been explored. The aim of this study was to explore the reported natural products of *G*. *sylvestre* against the RpfB of the Mtb. A total of 131 reported secondary metabolites of this plant were collected and virtually screened against the RpfB. We particularly targeted the Glu292 residue of RpfB as it is crucial for the catalysis of this protein. From our *in-house* library, 114 compounds showed a binding affinity higher than the standard drug. The binding stability of the top three lead compounds was further confirmed through MD simulation analysis. Drug likeness analyses indicated that the ten hits had zero violations of the Lipinski rule of five. In addition, analyses of pharmacokinetics, toxicity, and target prediction revealed that the top compounds are devoid of toxicity and do not affect human proteins. Additionally, they reflect multifaceted approach as anti-TB agents. Our selected hits not only exhibit molecular properties favoring physiological compatibility but also exhibit properties enhancing their potential efficacy as therapeutic candidates. The compounds investigated here are worthy of experimental validation for the discovery of novel treatments against TB. Further, this study also provides a promising avenue for research on the pharmacological potential of *G. sylvestre.*

## Introduction

*Mycobacterium tuberculosis* (Mtb) is the causative agent of one of the most prevalent infectious diseases, tuberculosis (TB). This pathogen mostly affects the lungs, but it can spread to other body parts such as the brain and spine (Chouhan et al., 2023). An important factor leading to its ineffective treatment is incorrect prescription guidance, causing Mtb to develop multi-drug resistance and increased activity in the infection process (Amorim et al., 2022). Mtb uses an effective survival strategy that alternates between active replication and latent non-replication. It goes into reversible low metabolic dormancy, allowing it to survive for a longer period of time without dividing (Kell and Young, 2000). Mtb is compromising the host’s immune defense mechanism due to its latent infections, resulting in immune system weakening (Rabaan et al., 2023), and lack of symptoms. According to a WHO study (2018) on the latent TB infection, Mtb affects over a third of the world’s population (WHO, 2021a), causing two million deaths annually (Corbett et al., 2003), mainly due to its dormancy and propensity to spread infection during this period (WHO, 2021a).

The increasing multi-drug resistance due to latent infections poses a life-threatening challenge, as current treatments such as isoniazid, rifampicin, ethambutol, and pyrazinamide are effective only for drug-susceptible TB (WHO, 2021b) and are inadequate for its multidrug-resistant, extensively drug-resistant, and totally drug-resistant strains (CDC, 2020; Rabaan et al., 2023). Hence, there exists an urgent necessity to comprehensively grasp the mechanisms underlying the dormancy period of Mtb. This understanding is pivotal to targeting its various metabolic pathways and restricting its strategy to enter low metabolic dormancy. Among the targets considered vital for bacterial growth, especially in the dormant period, is–resuscitation-promoting factor B (RpfB) (Chouhan et al., 2023). Resuscitation-promoting factors have five gene homologues, RpfA, RpfB, RpfC, RpfD, and RpfE, which help Mtb grow in the stationary phase. RpfB is the enzyme that is present in the cell wall of the bacteria and helps in dormancy. RpfB shows the highest complexity in its structure among the five homologs due to the presence of three DUF348 protein domains and one G5 domain, which is made up of two beta-sheets connected to a small triple helix motif (Ruggiero et al., 2016). Targeting the RftB protein is an effective strategy to treat TB as it interacts with peptidoglycan-hydrolyzing endopeptidase, controlling its activity and being responsible for the reactivation of Mtb in chronic cases (Hett et al., 2008).

Natural products extracted from plants have been used for the treatment of different diseases for a long time and are currently becoming more popular due to their low side effects (Aiman et al., 2018; Muhammad et al., 2021). Plants are rich in several antimicrobial secondary metabolites and may be a rich source of potent drugs with a variety of chemical moieties that could target different mechanisms in bacteria (Keita et al., 2022). Compounds like tomatidine showing promise as antibiotic potentiators, enhancing the effectiveness of antibiotics such as gentamicin, cefepime, and ciprofloxacin (Khameneh et al., 2019). Additionally, plant-based phytochemicals are being explored as alternatives to traditional antibiotics, offering new solutions to combat antimicrobial resistance (AlSheikh et al., 2020).

Traditionally used medicinal plants for treating TB includes the leaves of *Cercica papaya*, the seeds of *Combretum hereroense,* and roots of *Zanthoxylum capense.* Among the potential medicinal plants, *Gymnema sylvestre,* which belongs to the family of Apocynaceae, is traditionally used for the treatment of different disorders such as cardiovascular disorders, obesity, osteoporosis, asthma, and infectious disorders (Christopoulos et al., 2010; Tiwari et al., 2014). This plant possesses different medicinal activities, including antioxidant, antibiotic, anti-inflammatory, antiviral, gastroprotective, hepatoprotective, anticancer, and lipid-lowering properties (Khan et al., 2019). Despite the identification of different bioactive compounds from this plant, their possible role in the treatment of TB has not yet been explored. In this context, the aim of this study was to investigate the interacting ability of the reported secondary metabolites of *G. sylvestre* with the RpfB through molecular docking and molecular dynamic simulation studies. The druggability analysis of the lead inhibitors of RpfB opened a new avenue for the experimental validation of these compounds for the possible treatment of TB.

## Materials and methods

### Target protein selection and preparation

Resuscitation-promoting factor B (RftB), which revives dormant bacterial cells, is pivotal in TB research (Chouhan et al., 2023). The crystal structure of RpfB (PDB ID: 4EMN) was obtained from the Protein Data Bank (PDB). The selection of the 4EMN is justified by its direct relevance to MTb. Also, it depicts the RpfB catalytic domain in complex with benzamidine, obtained through X-ray diffraction. Its singular protein chain and high resolution of 1.17Å will provide a clear basis for precise docking simulations. This target protein was prepared using the Molecular Operating Environment (MOE) by removing water molecules and attached ligands to avoid any hurdles during protein-ligand interactions. It was energy minimized and protonated by using default parameters, and then saved as a MOE molecule file (Muhammad et al., 2020; Jaan et al., 2021).

### Active site preparation

The active sites of the RpfB were defined using the MOE Site finder tool. This tool predicted the pockets which are accessible for ligands to make interactions with target proteins. The best binding pocket was selected based on the position of the co-crystallized benzamidine and which is further supported by the reported active sites of RpfB (Chouhan et al., 2023). Dummy atoms were created to make the binding pockets approachable for the ligand atoms.

### Ligand selection and preparation

All the reported phytochemicals of *G. sylvestre* were selected as an in-house library for virtual screening against RpfB. The reported compounds were retrieved from the literature (Supplementary Table S1), drawn in ChemDraw 12.0 software, and saved in mol format. A ligand database was generated in the MOE using default parameters. Energy minimization of the ligand database was performed to keep the structures in an energetically favorable and stable conformation during docking. Benzamidine was used as a reference inhibitor in this study.

### Validation of the docking protocol

The validation step involves validating conformation by determining the RMSD value of the reference ligand conformation. Our docked RpfB-Benzamidine complex of the modeled structure and the crystallographically determined X-ray structure of RpfB (PDB ID: 4EMN) with co-crystalized benzamidine (PDB ID: BEN) were compared using the latter as a reference (Alananzeh et al., 2023). The docked protein-ligand complex was superimposed on the co-crystallized protein structure in PyMOL, and the output was set to calculate the RMSD of the docked ligands with the reference. RMSD value (≤2.0 A) was considered a successful docking protocol.

### Drug-likeness and physicochemical analysis

Further analysis of the compounds was performed by SwissADME (Daina et al., 2017). This web tool computes the drug-likeness, physicochemical, pharmacokinetic, and ADME properties. It also helps to predict the drugs-like nature and medicinal chemistry of compounds to support drug discovery. Different physicochemical properties of the compounds, i.e., molecular weight 150 g/mol < mw > 500 g/mol, H bond acceptors <10, H bond donors <5, polarity: 20 Å^2^ < and topological surface area (TPSA) < 160 Å^2^ were determined.

### Pharmacokinetic analysis

The pharmacokinetic features of the synthesized drugs were carefully examined in order to comprehensively evaluate their potential therapeutic value (Daina et al., 2017). The studied pharmacokinetics properties of the top compounds included blood-brain barrier permeation (BBB), human gastrointestinal absorption (GI), skin permeability parameters, plasma P-glycoprotein binding (P-gp), and interaction of the synthesized compounds with five important enzymes of the human cytochromes (P450), i.e., CYP1A2, CYP2D6, CYP2C19, CYP3A4, and CYP2C9. This thorough pharmacokinetic analysis provided essential details regarding how the compounds interact with biological systems, influencing their potential for future research and therapeutic use.

### Toxicity prediction

The cytotoxicity, hepatotoxicity, and LD50 values of the top compounds were Pevaluated using the ProTox-2 server (Banerjee et al., 2018).

### Target fishing analysis

Target-fishing analyses of the most promising compounds were performed to evaluate their interaction ability with human targets. For this purpose, the Swiss Target Prediction Server was used (Daina et al., 2019). The SMILES codes of the top compounds were generated and used as input files. The results were presented as scores ranging from 0 to 1, where the value 1 corresponds to the most likely target of the query molecule (Kamsri et al., 2019).

### PASS prediction

The top hits were subjected to PASS (Prediction of Activity Spectra for Substances) analysis (Filimonov et al., 2014) to find potential biological activities in addition to their predicted antibacterial potential for TB. The PASS server forecast the pharmacological effects, action mechanisms, and biological functions of molecules, based on the structure-activity relationships of established chemical compounds. It represents its predictions as probabilities, reflecting the chances of a compound exhibiting a certain biological activity (Pa). In this study, our attention was focused on properties with a Pa value exceeding 0.7, indicating a strong likelihood that our hits would demonstrate such activities. Given the abundance of the predicted properties, we specifically analyzed those common among the identified hits.

### Molecular docking, MD simulations and free energy calculation

In computational biology, molecular docking is an important technique for structure-based drug design to screen potential targets, allowing the calculation of the binding energy of the drug-target complexes and monitoring their reliable interactions at the atomic level (Shah et al., 2021). Molecular docking of RpfB against the energy-minimizing ligand database was done by computing the DOCK command in MOE. The induced fit behavior of the receptor was employed to evaluate the flexible docking of the receptor molecule. Dummy atoms were selected from the sites module; they participated as protein active site during the molecular docking process. Five conformational poses were generated in the output results, and the highest-scored pose of each ligand based on the “S” score and binding energy was considered. The scores of ligands were compared with those of benzamidine as the reference inhibitor. Ligands with a higher docking score than the reference compound were shortlisted for further investigations.

The top three ranked compounds after preliminary analysis were subjected to MD simulation for detailed analysis. The protein was used as receptors, and the top-ranked compounds were prepared as ligands as per the standard protocol of GROMACS (Aslam et al., 2021; Sarfraz et al., 2023). After nvt and npt analysis, the constructed complex was subjected to a production run of 200 ns, and results were analyzed using vmd and in-house scripts (Van Der Spoel et al., 2005).

Moreover, Alchemical free energy simulations have been used for the prediction of free energy changes to explain the binding and unbinding mechanism of the three top hits. The binding free energy (dG_binding_) was determined using standard protocol of NAMD (Kim et al., 2020; Phillips et al., 2020). The initial files and simulations step up was established using online server *Free Energy Calculator* (http://www.charmm-gui.org/input/fec). The chemical free energy perturbation for absolute ligand binder was chosen for molecular dynamic simulations (Oshima et al., 2020).

$$\Delta G_{\text{binding}}=\Delta G_{\text{Ligand}}-\Delta G_{\text{complex}}$$

## Results and discussion

### Validations of docking methodology

**FIGURE 1:**
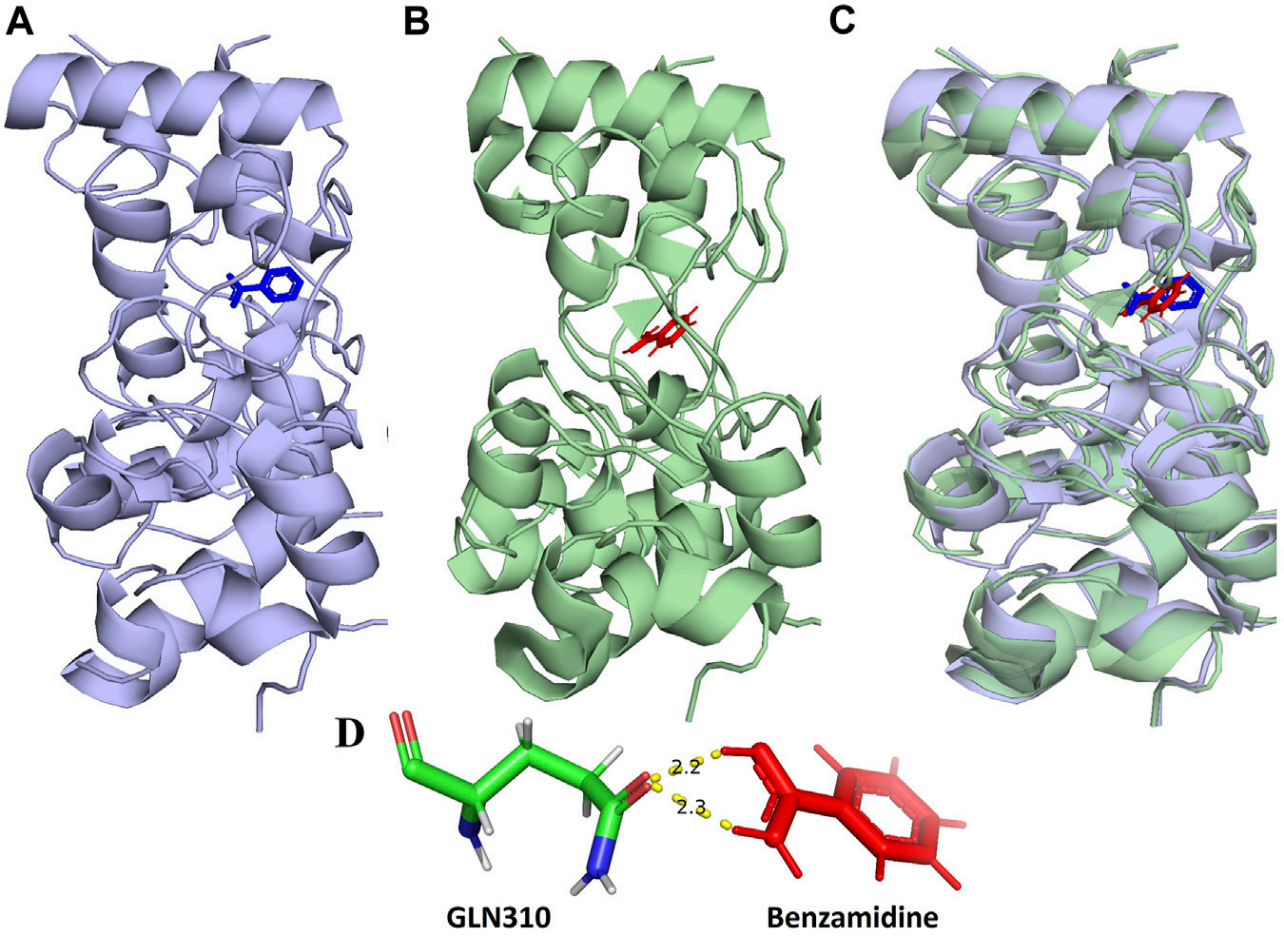
**(A)** Show the location of the binding of benzamidine (blue) in the 3D structure of RpfB (4EMN). **(B)** Binding location of the re-docked benzamidine (red) in RpfB **(C)** Superimposition of A and B complexes shows binding of benzamidine at the same location. **(D)** Molecular interaction of benzamidine in the re-docked benzamidine-RpfB complex.

The validation of docking methodology was carried out between the co-crystallized Benzamidine-RpfB complex (PDB ID: 4EMN) and the re-docked Benzamidine-RpfB complex. This procedure involved the comparison of 322 residues from both structures. During this alignment, we were able to achieve a match align score of 1780.000, indicating a high level of congruence between the two structures. Notably, in the rigorous refinement process, we observed the exclusion of a small subset of atoms to achieve a more accurate alignment. Specifically, 10 atoms were rejected in the first cycle, followed by the rejection of 3 atoms in the second cycle. Ultimately, the final alignment resulted in the low RMSD value of 0.967 Å, calculated across 309 atoms. This value not only reflects the high quality of our alignment but also serves as a validation of our docking protocol, underscoring its reliability and accuracy in structural comparisons. The superimposition of these structures showed that they share similar binding pockets, suggesting that the ligands engage in analogous molecular interactions across these targets (Figures 1A–C).

### Molecular docking results

Molecular docking was accomplished with 129 secondary metabolites of the therapeutic herb *G. sylvestre* and the RpfB protein of Mtb. Binding energies were calculated for all the compounds and ranked accordingly to select the top hits (Supplementary Table S1). The reference ligand showed a docking score of −4.1 kcal/mol. Here, it was found that 110 compounds had a higher docking score than the reference. The docking scores of the top 60 compounds were in the range of −8.25 kcal/mol to −5.1 kcal/mol (Supplementary Table S1), while those of the top ten in the range of −6.2 to −5.1 kcal/mol (Supplementary Table S2). From the results of molecular docking, it was observed that ten compounds, namely, Hop −22 (29)-en-3β-ol, Dimethy 2-methoxy hexane, Benzazirene Carboxylic Acid, Calcium Oxalate, Gymnestrogenin, Lupeol, Gymnemoside C, Gymnemoside D, Gymnemic Acid VII, and Gymnemanol, were inactive and did not show any kind of interactions with the RpfB of Mtb (Supplementary Table S1), This might be due to the structural complexity or flexibility of these compounds, which can hinder the generation of a suitable conformation for docking. Additionally, these compounds may not have been compatible with the chosen binding site, resulting in unsuccessful docking. It is clear that these ten compounds are ineffective as potential inhibitors for RpfB since they are unable to successfully interact with the target protein. This implies that additional structural modification of these particular compounds may be necessary to increase their binding affinity.

### Protein-ligands interactions

**FIGURE 2:**
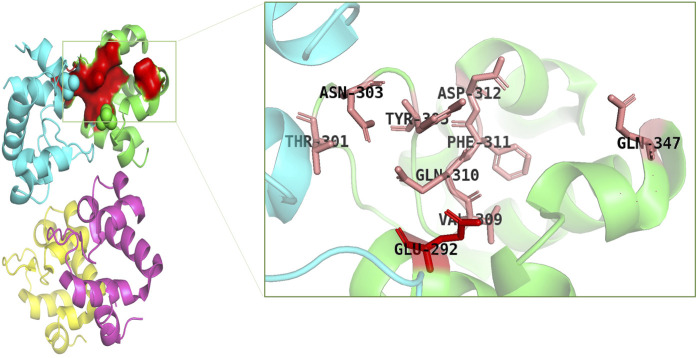
Catalytic pocket of RpfB (4EMN); catalytically significant residues are highlighted in pink, and the central residue is highlighted in red.

Catalytically significant residues of Rpfb, i. e., Glu292, Tyr305, Val309, Gln310, Phe311, Asp312, Thr301, Gln347 and along with the hydrophobically interacting residues, i. e., CYS291, ILE299, ASN300, THR301, GLY304, TYR305, TYR306, GLY307, GLN310, GLY350, ALA350, PRO354 and CYS355were considered in the active site (Figure 2). It has been observed that residues, particularly Glu292, play an essential role in the hydrogen bond interactions, thereby stabilizing the inhibitors in the catalytic pocket. Moreover, Glu292 is the only amino acid residue that is crucial and indispensable in the catalytic activity and is responsible for the RpfB functions (Squeglia et al., 2013). Interaction with Gln310 is also significant in inhibition, as benzamidine binds to only this residue to inhibit RpfB (Rabaan et al., 2023), and this study also supported these results (Figure 1D).

**FIGURE 3:**
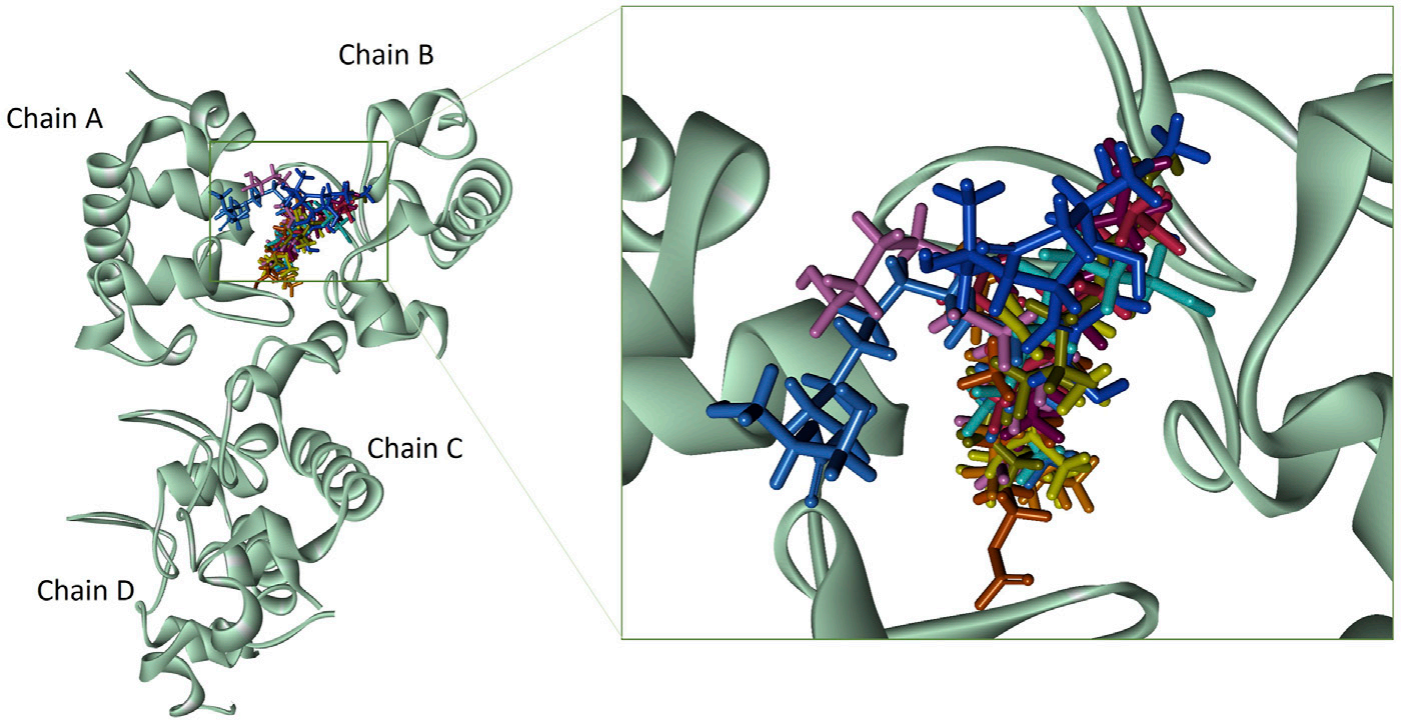
Binding of top compounds in the catalytic pocket of RpfB The catalytic pocket resides between chains A and B: nerolidol (yellow), dodecanol (orange), benz(e)azulene-3, 8-dione (zinc-blue), 2-pentadecanone (dark pink), 2-palmitoglycerol (blue), tetradecanoic acid (army green), 6-Octen-1-ol, 3,7-dimethylformate (brown), 8-dodecenol (cyan), methyl tetra decanoate (purple), and tetradecenol (light pink).

**FIGURE 4:**
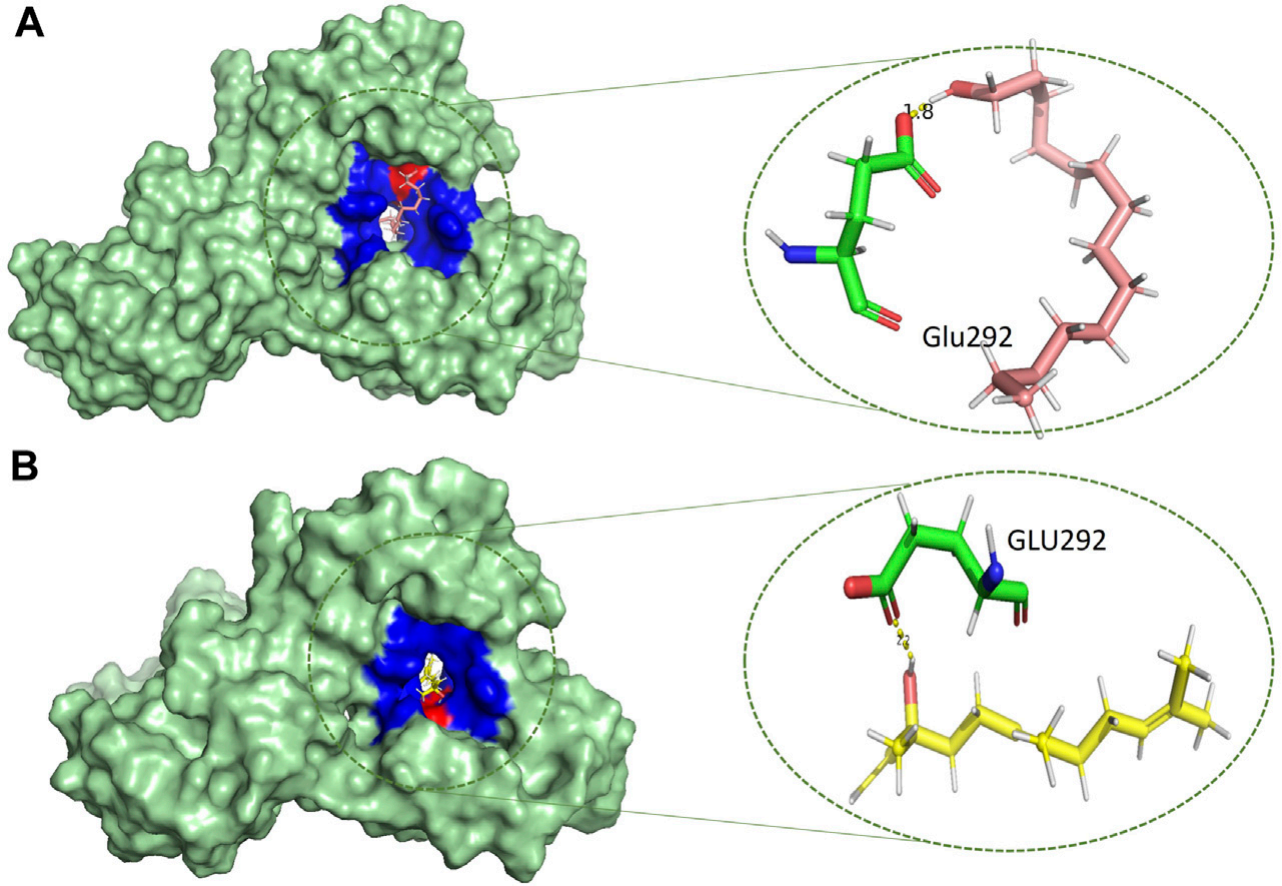
Molecular interactions (light green) of **(A)** tetradecanol (light pink) and **(B)** nerolidol (yellow) with RpfB (green), with distinct representations of hydrophilic (red) and hydrophobic (blue) interactions.

**FIGURE 5:**
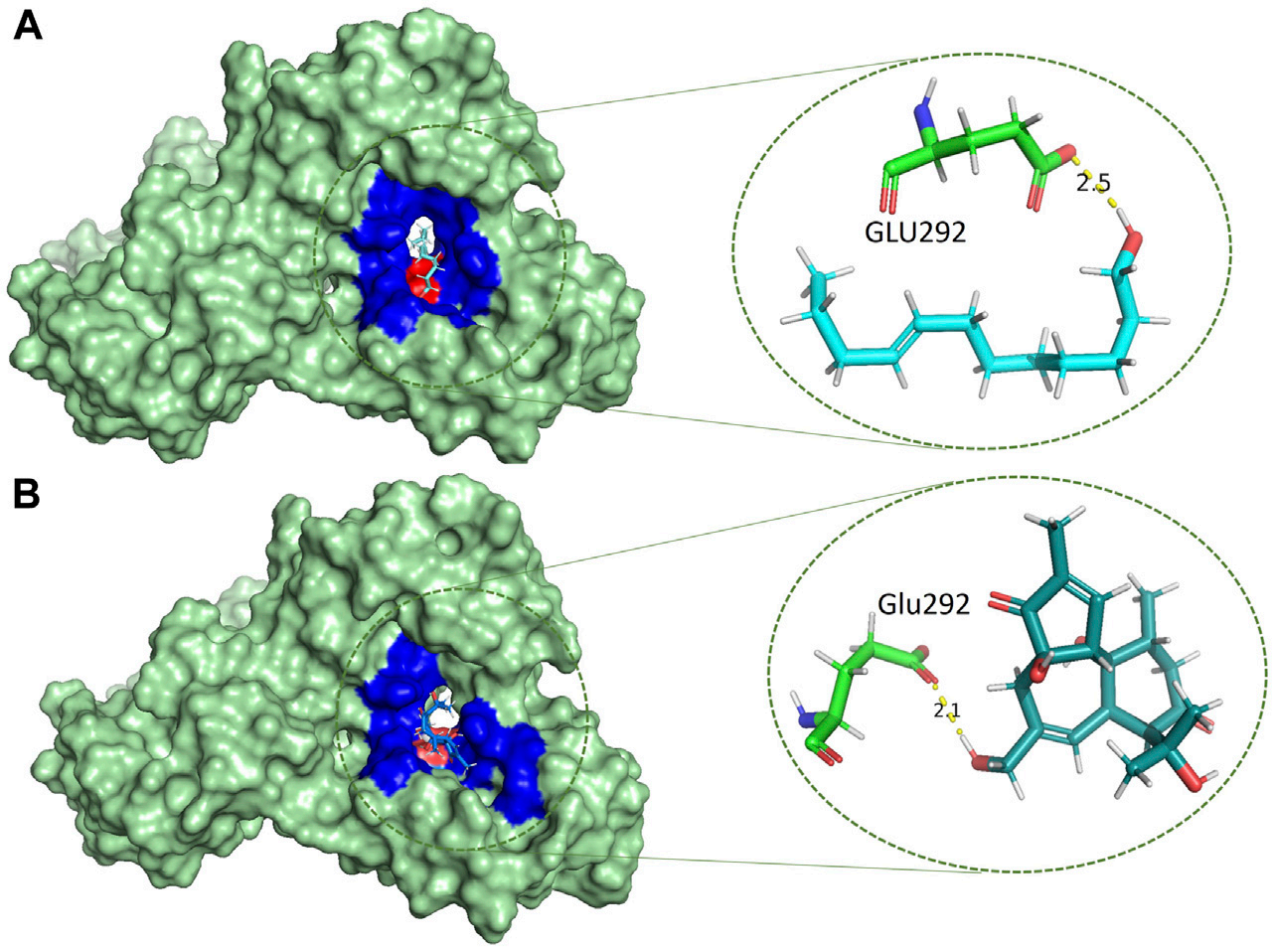
Molecular interactions (light green) of **(A)** 8-Dodecenol (cyan) and **(B)** Benz(e)azulene-3,8-dione (zinc-blue) with RpfB (green) with distinct representations of hydrophilic (red) and hydrophobic (blue) interactions.

Based on the drug-likeness criterion, ten ligands were selected for interactions analysis with the receptor. These compounds include 2-Palmitoglycerol, Benz(e)azulene-3,8-dione, Nerolidol, 1-Dodecanol, Tetradecanoic acid, 6-Octen-1-ol-3,7-dimethylformate, 8-Dodecenol, Tetradecanol, Methyl tetra decanoate, and 2-Pentadecanone (Supplementary Table S2). All of the top hits bound to the same region in the catalytic pocket between chains A and B (Figure 3). This position of the hits is similar to the binding location of benzamidine (Figures 1A,B). We observed the hydrogen bond interactions of the ten hits mainly with Glu292 and Gln310 (Supplementary Table S2). The limited interaction with other residues in the active site suggests a highly targeted mechanism. This focused interaction pattern emphasizes the importance of these residues in the binding affinity and specificity of the compounds. The N-acetyl glucosamine moiety of the Mtb cell wall shows similar interactions with Glu292 and Gln310 of RpfB. This interaction (between RpfB and Mtb cell walls) is involved in the processes that support growth, division, and the maintenance of cell wall integrity (Squeglia et al., 2013). Thereby, targeting these crucial residues significantly suppresses RpfB activity. Some compounds interact with the RpfB protein with shorter bond lengths which indicate stronger bond formation between the protein and the ligands. One of the hits, Tetra-decanol, exhibited hydrogen bonding to the Glu292, with the bond length of 1.8Å (Figure 4A), suggesting biologically significant binding in the inhibition of RpfB. Tetradecanol has been reported previously as an antimicrobial agent (Dherbomez et al., 2006). Other hits showing molecular interaction with RpfB by forming one hydrogen bond with the functional residue, Glu292, are Nerolidol (Figure 4B), 8-Dodecanol (Figure 5A) and Benz(e)-azulene-3,8-dione (Figure 5B) with 2.2Å, 2.5Å and 2.1Å, respectively. Among these compounds, Nerolidol has previously been reported for its antimicrobial, pesticidal, and anti-acne properties (Srinivasan and Kumaravel, 2016).

**FIGURE 6:**
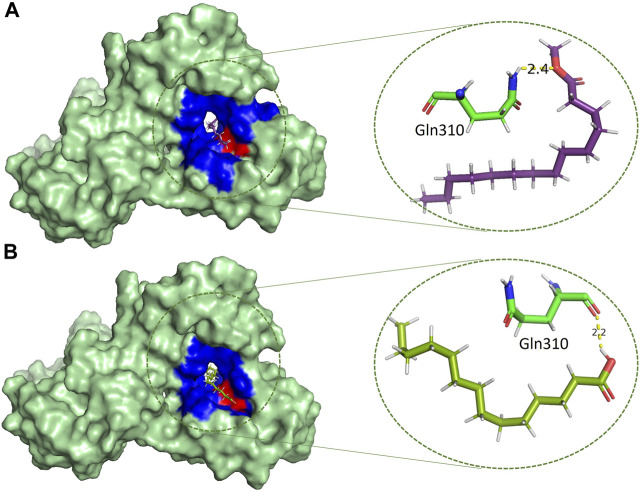
Molecular interactions (light green) of **(A)** methyltetradecanoate (purple) and **(B)** tetradecanoic acid (army-green) with RpfB (green), with distinct representations of hydrophilic (red) and hydrophobic (blue) interactions.

Methyl tetra decanoate (Figure 6A) and Tetradecanoic acid (Figure 6B) formed a single hydrogen bond with the GGln310, similar to the reference compound Benzamidine with a bond length of 2.4 Å and 2.2 Å, respectively. Tetradecanoic acid has been reported to have antioxidant, anticancer and hypercholesterolemic activities (Srinivasan and Kumaravel, 2016). 2-Pentadecanone showed interactions with the Asn303 of the protein with a bond length of 2.3 Å (Supplementary Figure S1A). 2-pentadecanone has been reported to have wound healing and the antibacterial activity (Mag et al., 2019). 2-Palmitoglycerol, one of the hits, forms a hydrogen bond with Asp312 of the catalytic site of Rpfb (Supplementary Figure S1B). Asp312 is responsible for a more negatively charged electrostatic potential in the substrate binding cleft of RpfB, and it may be associated higher enzymatic activity, but Asp312 is not conversed in all Mtb homologues (Squeglia et al., 2013). Binding between 2-Palmitoglycerol and RpfB could be significant in suppressing RpfB activity. 2-Palmitoglycerol has previously been reported as an antibacterial agent (Adnan et al., 2019). 6-Octen-1-ol-3,7-dimethylformate (Supplementary Figure S2B) formed a hydrogen bond with a minimum bond length of 1.9Å with the Thr301.6-Octen-1-ol-3,7-dimethyl-formate has previously shown monoterpenoid-antiallergenic antimicrobial, and pesticide activity (Srinivasan and Kumaravel, 2016). 1-Dodecanol is the only compound that makes four hydrogen bonds with the RpfB protein with different amino acid residues such as Asn303, Gly302, Thr301 and Tyr305, with different bond lengths (Supplementary Figure S2B). 1-Dodecanol has antibacterial potential and is an insecticide permitted as a food additive in both the U.S. and EU (Togashi et al., 2007; Kazek et al., 2021).

The residues involved in hydrophobic interaction with ligands were mostly common and CYS291, ILE299, ASN300, THR301, GLY304, TYR305, TYR306, GLY307, GLN310, GLY350, ALA350, PRO354, and CYS355. Interestingly, the hydrophobic pocket of RpfB made by these residues is well conserved in the other resuscitation-promoting factors. The preservation of this pocket in all resuscitation-promoting factor types, i.e., A-E, suggests that a hydrophobic environment for Glu292 is important for the catalytic mechanism of these enzymes. Similar to lysozymes and lytic transglycosylases, this hydrophobic environment likely increases the pKa of Glu292, thus allowing this residue to engage a hydrogen-bonding interaction with the glycosidic oxygen of the scissile bond (Inoue et al., 1992). All the significant hydrophobic interactions were observed with the ten hits except for PRO354 (Supplementary Table S2). The maximum number of interactions with the hydrophobically significant residues were observed with Tetradecanoic acid and 2-pentadecanone (Supplementary Table S2). All the lead hits were found to be engaged in hydrophobic interactions in the RpfB binding site (Figures 4–6, Supplementary Figures S1, S2); while they formed 1 to 3 hydrogen bonds, the hydrophobically interacting residues were 8–13. This phenomenon arises from the essential hydrophobic function performed by the active site residues of RpfB. These hydrophobic interactions are crucial for RpfB to bind to its substrates and perform its enzymatic activities; also, hydrophobic interactions support hydrogen bonds with the catalytically significant residues of RpfB (Inoue et al., 1992).

### Drug-likeness and physicochemical analysis

Based on their binding energies, the top sixty compounds were subjected to drug-likeness and physiochemical analysis through Swiss ADME (Daina et al., 2017). It was observed that twenty two compounds did not follow any drug-like property due to their excessive molecular weight (>700 gmol^-1^). Ten compounds were narrowed down from the pool of remaining thirty-eight compounds for thorough examination (as described in the context of molecular interaction analysis), namely, 2-Palmitoglycerol, Benz(e)azulene-3,8-dione, Nerolidol, 1-Dodecanol, Tetradecanoic acid, 6-Octen-1-ol-3,7-dimethylformate, 8-Dodecenol, Tetradecanol, Methyl tetra decanoate, and 2-pentadecanone. These compounds were selected due to their druggability potential and following the Lipinski criterion with no violations, suggesting a promising possibility of having desirable drug-like properties (Lipinski et al., 1997). The compounds meeting this requirement showed adherence to characteristics, including molecular weight (MW), count of hydrogen bond acceptors and donors, logarithm of the partition coefficient (Log P), logarithm of solubility (Log S), molar refractivity (MR), topological polar surface area (TPSA), and potential for gastrointestinal absorption (Table 1). This suggests a favorable balance between hydrophilicity and lipophilicity, ensuring adequate absorption and distribution within the body. The MW, hydrogen bonding capacity and Log P collectively inform on the compound’s ability to penetrate cell membranes, while Log S, MR and TPSA are indicative of solubility and overall molecular interaction potential (Ferreira and Andricopulo, 2019).

**TABLE 1:** Drug likeness and physicochemical analysis of the compounds showing higher docking score than benzamidine.

Sr. No.	Compound	Molecular formula	Molecular weight (g/mol)	HA	HBA	HBD	Log P	Log S	MR	TPSA 99 (A^2)	GI absorption	Lipnski rule
1	Vitamin E	C_29_H_50_O_2_	430.71	31	2	1	5.92	−8.60	139.27	29.46	Low	Yes; 1violation
2	Nerolidol	C_15_H_26_O	222.37	16	1	1	3.64	−3.80	74.00	20.23	High	Yes
3	Oleic acid	C_18_H_34_O_2_	282.46	20	2	1	4.01	−5.41	89.94	37.30	High	Yes; 1 violation
4	Ergosterol	C_28_H_44_O	396.65	29	1	1	4.01	−6.72	127.47	20.23	Low	Yes; 1violation
5	Cholestan 3 one	C_27_H_46_O	386.65	28	1	0	4.07	−7.55	123.13	17.07	Low	Yes; 1 violation
6	Dodecanol	C_12_H_26_O	186.33	13	1	1	3.37	−3.57	60.96	20.23	High	Yes
7	2-pentadecanone	C_15_H_30_O	226.40	16	1	0	4.01	−4.40	74.42	17.07	High	Yes
8	Hexahydrofarnesyl	C_18_H_36_O	268.48	19	1	0	4.39	−5.09	88.84	17.07	High	Yes; 1 violation
9	Ethyl palmitate	C_18_H_36_O2	284.48	20	2	0	4.65	−5.51	89.92	26.30	High	Yes; 1 violation
10	9,12,15-octadecatrienal	C_18_H_30_O	262.43	19	1	0	4.06	−4.64	87.42	17.07	High	Yes; 1 violation
11	Cholane-5,20 (22)-diene-3b-phenoxy	C_30_H_42_O	418.65	31	1	0	5.15	−8.03	133.57	9.23	Low	Yes; 1violation
12	1-pentadecanol	C_15_H_32_O	228.41	16	1	1	4.22	−4.54	75.38	20.23	High	Yes; 1 violation
13	Hopeaphenol	C_56_H_42_O_12_	906.93	68	12	10	2.69	−11.40	252.52	220.76	Low	No; 3 violations
14	2-palmitoglycerol	C_19_H_38_O_4_	330.50	23	4	2	4.50	−4.57	97.06	66.76	High	Yes
19	Tetratriacontane	C_34_H_70_	478.92	34	0	0	8.92	−12.12	165.55	0.00	Low	Yes; 1 violation
20	Triacontane	C_30_H_62_	422.81	30	0	0	7.86	−10.68	146.32	0.00	Low	Yes; 1 violation
21	Alpha tocopherol acetate	C_31_H_52_O_3_	472.74	34	3	0	6.28	−8.78	148.75	35.53	Low	Yes; 1 violation
22	Diheptan-3-yl Benzene 1,2 Dicarboxylate	C_22_H_34_O_4_	362.50	26	4	0	4.80	−5.58	106.69	52.60	High	Yes; 1 violation
23	Hexacosane	C_26_H_54_	366.71	26	0	0	7.08	−9.23	127.10	0.00	Low	Yes; 1 violation
24	Gamma tocopherol	C_28_H_48_O_2_	416.68	30	2	1	5.16	−8.29	134.31	29.46	Low	Yes; 1 violation
25	Phytyl acetate	C_22_H_42_O_2_	338.57	24	2	0	5.13	−6.47	108.68	26.30	Low	Yes; 1 violation
27	Isophytol	C_20_H_40_O	296.53	21	1	1	4.88	−5.75	98.98	20.23	Low	Yes; 1 violation
29	Pentacosane	C_25_H_52_	352.68	25	0	0	6.81	−8.87	122.29	0.00	Low	Yes; 1 violation
30	Ethyl Octadec-9-enoate	C_20_H_38_O_2_	310.51	22	2	0	5.03	−5.70	99.06	26.30	Low	Yes; 1 violation
31	Eicosane	C_20_H_42_	282.55	20	0	0	5.64	−7.05	98.25	0.00	Low	Yes; 1 violation
32	A-Norcholestan-3-one, 5-ethenyl	C_28_H_46_O	398.66	29	1	0	4.89	−7.69	127.20	17.07	Low	Yes; 1 violation
34	Methyl palmitate	C_17_H_34_O_2_	270.45	19	2	0	4.41	−5.18	85.12	26.30	High	Yes; 1 violation
35	Bicyclo [2.2.1]heptane, 1,3,3-trimethyl	C_22_H_32_N_2_O_4_S	420.57	29	5	1	3.52	−4.46	117.53	84.09	High	Yes
36	Stigmasterol	C_29_H_48_O	412.69	30	1	1	5.08	−7.46	132.75	20.23	Low	Yes; 1 violation
50	Methyl tetra decanoate	C_15_H_30_O_2_	242.40	17	2	0	75.06	3.88	−4.52	26.30	High	Yes
51	1- pentadecanol	C_15_H_32_O	228.41	16	1	1	75.38	4.22	−4.54	20.23	High	Yes; 1 violation
53	Tetradecanoic acid	C_14_H_28_O_2_	228.37	16	2	1	71.18	3.32	−4.31	37.30	High	Yes
54	8-dodecanol	C_12_H_24_O	184.32	13	1	1	60.49	3.45	−3.02	20.23	High	Yes
55	Taxasterol	C_50_H_30_O	426.42	31	1	1	135.14	4.68	−8.02	20.23	Low	Yes; 1 violation
56	Beta elemene	C_15_H_24_	204.35	15	0	0	70.42	3.37	−4.76	0.00	Low	Yes; 1 violation
57	6-Octen-1-ol, 3,7-dimethyl-, formate	C_11_H_20_O_2_	184.28	13	2	0	36.19	2.84	−3.36	56.19	High	Yes
58	Tetradecenol	C_14_H_300_	214.39	15	1	1	70.57	3.90	−4.18	20.23	High	Yes
59	Benz(e)azulene-3,8-dione	C_20_H_28_O_6_	364.43	26	6	4	96.13	0.41	−1.68	115.06	High	Yes

It is noteworthy that, we opted for the strict adherence to Lipinski’s Rule of Five to ensure the selection of compounds with optimal drug-like properties. While it is acknowledged that certain antibiotics in the market may not strictly adhere to these criteria, our choice was guided by the desire to prioritize compounds with favorable pharmacokinetic profiles. Nevertheless, the efficacy of antibiotics can be multifaceted, and deviations from these rules in real-world scenarios underline the complexity of antibiotic drug design. This meticulous process guarantees that the selected compounds not only exhibit molecular properties favoring physiological compatibility but also exhibit properties enhancing their potential efficacy as therapeutic candidates. By combining these multifaceted parameters, the ten compounds emerge as prime candidates warranting in-depth evaluation for their therapeutic potential (Table 1).

### Pharmacokinetic analysis

Pharmacokinetic analysis such as ADME plays a central role in drug design before the clinical trial and decreases the chances of drug failure in the clinical trial (Pires et al., 2015). A compound that has GI absorption >30% shows better absorbance in the body. All hits were predicted to possess high GI absorptions, and they were predicted to not be P-gp (glycoprotein) substrates except Benz(e)-azulene-3,8-dione, providing their favorable intestinal absorption (Table 2). The P-gp is known for efflux of a wide range of molecules, including drug substances, as this can lead to reduced bioavailability or increased resistance (Dahlgren and Lennernäs, 2019). All hits along the reference compound demonstrated the noteworthy ability to permeate the blood-brain barrier (BBB). To mitigate potential neurotoxicity risks of the hits capable of crossing the BBB, we propose structural modifications to reduce BBB permeability and early-stage predictive toxicity screenings. Targeted drug delivery systems can also be employed to limit CNS exposure.

**TABLE 2:** Pharmacokinetic analysis of top compounds performed with the Swiss ADME server.

Sr. No.	Compound name	GI absorption	BBB permeation	P-gp substrate	CYP142 inhibitor	CYP2C19 inhibitor	CYP2C9 inhibitor	CYP2D6 inhibitor	CYP3A4 inhibitor
1	Nerolidol	High	Yes	No	Yes	No	Yes	No	No
2	Dodecanol	High	Yes	No	Yes	No	No	No	No
3	2-Pentadecanone	High	Yes	No	Yes	No	No	No	No
4	2-Palmitoglycerol	High	Yes	No	No	No	No	Yes	No
5	Tetradecanoic acid	High	Yes	No	Yes	No	No	No	No
6	6-Octen-1-ol-3,7-dimethyl- formate	High	Yes	No	No	No	Yes	No	No
7	8-Dodecenol	High	Yes	No	Yes	No	No	No	No
8	Methyltetradecanoate	High	Yes	No	Yes	No	No	No	No
9	Tetradecanol	High	Yes	No	Yes	No	No	No	No
10	Benz(e)-azulene-3,8-dione	High	No	Yes	No	No	No	No	No
Standard	Benzamidine	High	Yes	No	No	No	No	No	No

Benz(e)-azulene-3,8-dione and the reference compound benzamidine showed no cytochrome inhibitory potential. These enzymes are responsible for 90% of drug metabolism as well as interfering with the metabolism of a variety of endogenous substances (Kamsri et al., 2019). This suggests that these compounds would not have restrictions due to the inhibition of these cytochromes and would not be metabolized in the body. According to the predictions, CYP142 inhibitors included Nerolidol, 1-Dodecanol, 2-Pentadecanone, Tetra-decanoic acid, 8-Dodecenol, Methyl tetra decanoate and Tetra-decenol. Nerolidol and 6-Octen-1-ol-3,7-dimethylformate can inhibit CYP2C9 cytochromes. CYP2C19 and CYP3A4 were not predicted to be inhibited by any of the hits. This suggests that all ten hits have low affinity for the active sites of two major cytochromes, i.e., CYP2C19 and CYP3A4. Therefore, these compounds will mostly be exposed to CYP2C9, CYP142, and CYP2D6 during absorption, distribution, metabolism, and excretion, and there will be minimal exposure to CYP2C19 and CYP3A4 enzymes during these processes. This might be achieved through rapid clearance or specific tissue targeting.

### Cytotoxicity, hepatotoxicity and LD50 value analysis

The prediction of the toxicity of the top ten hits was performed by a ProTox II server (Banerjee et al., 2018). All the hits were predicted to be non-cytotoxic; they show more than a 74% probability of not being cytotoxic agents (Supplementary Table S3). Hepatotoxicity analyses revealed that Tetradecanoic acid has a 52% probability of not being a hepatotoxic agent. LD50 values showed that all the compounds lie in the non-toxic classes except Benz(e)-azulene-3,8-dione which has a LD50 value of 50 kg/mol, and may be toxic to some extent if swallowed (Supplementary Table S3). The non-toxic nature of top hits ensures patient safety and the quality of their lives by minimizing the risk of adverse reactions and harmful effects in the body. Moreover, our hits are more likely to progress efficiently through preclinical studies and clinical efficiently trials, reducing development costs and failures.

### Target fishing analysis

Target fishing analyses were performed to identify possible human targets for the selected hits, with a probability value ranging from 0 to 1 (Galati et al., 2021). This step is crucial in assessing the comprehensive pharmacodynamics profile of the hits, particularly for treating Mtb. We aimed to predict both beneficial and harmful interactions within the body. Understanding these interactions helps in evaluating the drug’s efficacy and safety, as they can influence both the disease pathology and the drug’s therapeutic potential.

The top leads were analyzed by SWISS target prediction to determine if they might inhibit any human protein (Table 3). Among our top hits only one compounds, i.e., Tetradecanoic acid show a high probability (85%) of interacting with human targets, including peroxisome proliferator-activated receptors and fatty acid binding proteins. These targets are involved in lipid metabolism and regulation, thereby suggesting the role of Tetradecanoic acid in such processes (Table 3). Other than Tetradecanoic acid, all other hits showed a very low probability interacting with the human targets. This is significant in terms of the safety and specificity of these compounds. It greatly reduces the risk of adverse effects on human cells and minimizes the potential for drug-drug interactions when used in combination with other medications. 2-Palmitoglycerol, Tetradecenol, 8-Dodecenol and 1-Dodecanol were predicted to interact with various receptors and enzymes, but with very low probabilities (8%–16%). Most of these receptors and enzymes are related to signal transduction and metabolism. Notable targets include protein kinase C alpha, transient receptor potential channels, and CYP2C19. Methyltetradecanoate and 2-pentadecanone were predictively linked to carbonic anhydrase I and II but with very low probabilities of 0.2 and 1.2, respectively, suggesting a limited role in modulating carbon dioxide transport and pH regulation with very low probabilities. 6-Octen-1-ol-3,7-dimethylformate predicted interactions with receptors like serotonin and adrenergic receptors indicating limited involvement in neurotransmission and hormonal signaling. The least possible interactions of Nerolidol with Squalene monooxygenase and Indoleamine 2,3-dioxygenase point toward its non-existent roles in cholesterol synthesis and immune regulation. There were no human targets predicted for Benz(e)-azulene-3,8-dione (Table 3). This suggests that Benz(e)-azulene-3,8-dione will not interact with any human target when taken as an antibiotic drug. This suggests the high degree of the specificity of Benz(e)-azulene-3,8-dione towards the RpfB of the MTb, potentially reducing the likelihood of off-target effects and adverse reactions in the human body. Such specificity could translate into favorable safety profile, making Benz(e)-azulene-3,8-dione a promising candidate for further development. These results demonstrated that our hits can be used across a broader spectrum of patients, regardless of individual variations in human targets, making them versatile and valuable in the treatment of infectious diseases.

**TABLE 3:** Target fishing analysis of top compounds by swiss target prediction tool.

Sr. No.	Predicted targets	Probability
1	2-Palmitoglycerol	
	Protein kinase C alpha	0.12
	Peptidyl-glycine alphaamidating Monooxygenase	0.10
	NAD-dependent deacetylase sirtuin 2	0.10
	Protein-tyrosine phosphatase 1B	0.10
	Protein kinase C gamma	0.10
2	Methyl tetra decanoate	
	Carbonic anhydrase II	0.2
	Carbonic anhydrase I	0.2
	Estradiol 17-betadehydrogenase 3	0.05
	Fatty acid binding protein adipocyte	0.04
	Peroxisome proliferator activated receptor alpha	0.04
3	Tetra-decenol	
	Transient receptor potential cation channel subfamily M member 8	0.12
	Carbonic anhydrase II	0.09
	Carbonic anhydrase I	0.09
	Carbonic anhydrase IV	0.09
	Androgen Receptor	0.08
4	6-Octen-1-ol-3,7-dimethylformate	
	Transient receptor potential cation channel subfamily A member 1	0.07
	Cholesterol esterase	0.07
	Serotonin 2b (5-HT2b) receptor	0.07
	Alpha-2a adrenergic receptor	0.07
	Serotonin 2c (5-HT2c) receptor	0.07
5	2-pentadecanone	
	Carbonic anhydrase I	0.12
	Carbonic anhydrase II	0.12
	Acyl coenzyme A: cholesterol acyltransferase	0.10
	Carboxylesterase 2	0.10
	Nuclear receptor subfamily 1 group I member 3 (by homology)	0.09
6	8-Dodecenol	
	Nuclear receptor subfamily 1 group I member 3 (by homology)	0.16
	Cytochrome P450 2C19	0.14
	Androgen Receptor	0.12
	Norepinephrine Transporter	0.09
	Protein-tyrosine phosphatase 1B	0.09
7	Tetradecanoic acid	
	Peroxisome proliferator activated Receptor alpha	0.85
	Fatty acid binding protein muscle	0.55
	Free fatty acid receptor 1	0.55
	Peroxisome proliferator activated receptor delta	0.54
	Fatty acid binding protein adipocyte	0.51
8	Dodecanol	
	Transient receptor potential cation channel subfamily M member 8	0.16
	Testis specific androgen-binding protein	0.13
	Dual specificity phosphatase Cdc25B	0.13
	Dual specificity phosphatase Cdc25A	0.12
	Androgen Receptor	0.12
9	Nerolidol	
	Squalene monooxygenase	0.11
	Indoleamine 2,3- dioxygenase	0.06
	Period circadian protein homolog 2	0.06
	Beta-secretase 1	0.06
	Glucagon receptor	0.06

### Prediction of Activity Spectra

We employed the PASS prediction analysis based on a stringent selection criteria, ensuring that only those properties were considered that showed more than 0.7% probability. Despite the multitude of properties, we strategically focused on commonly shared among the hits. Such analysis is crucial in highlighting alternative therapeutic attributes beyond their primary antibiotic action, possible uncovering multifaceted roles in TB management.

Most of the properties were shown by Tetradecanoic acid and 8-Dodecenol (Supplementary Table S4). The anti-inflammatory property predicted for the hits, is crucial given the inflammatory response often seen in TB infections, which can exacerbate patient suffering and disease progression (Piergallini and Turner, 2018). The anti-hypoxic property is another key finding, given the ability of MTb to survive in hypoxic conditions within the host (Jakkala and Ajitkumar, 2019). Also, as hypoxic Mtb cells have been found to be resistant to anti-tuberculosis drugs, compounds exhibiting this property could play a crucial role in targeting the bacteria in these challenging environments, potentially enhancing the efficacy of TB treatments.

The anti-eczematic and anti-seborrheic properties of most of the hits, while not directly related to TB, indicated a broader spectrum of dermatological benefits, potentially reducing side effects or co-morbid conditions in TB patients (Siewchaisakul et al., 2021). This aspect is particularly relevant given the prolonged treatment regimens required for TB, which often result in patient non-compliance due to adverse effects.

2-Palmitoglycerol was also predicted to have anti-infective and antitoxic property with high probability. Its anti-infective activity suggests its potential in preventing or reducing infections, a critical aspect in TB treatment where secondary infections can complicate patient recovery (Moule and Cirillo, 2020). While the antitoxic capability of 2-Palmitoglycerol indicates its role in neutralizing toxins or reducing their harmful effects. In TB, where the bacterial toxin can contribute to disease severity, this property could provide a dual approach to treatment, targeting both the bacteria and the toxins produced (Pajuelo et al., 2021). Moreover, Benz(e)azulene-3,8-dione due to its unique structure among hits revealed a spectrum of bioactive properties that could be instrumental in developing comprehensive treatment strategies. These properties included its anti-parasitic and anti-helmintic activity. The combined properties of these compounds highlight their potential as multi-target agents in the treatment of TB and associated conditions.

In conclusion, the PASS predictions have provided valuable knowledge about the potential of our hits as anti-TB agents and additional beneficial properties. Further experimental validation of these predictions is necessary to confirm their efficacy and safety.

### MD simulation trajectories

**FIGURE 7:**
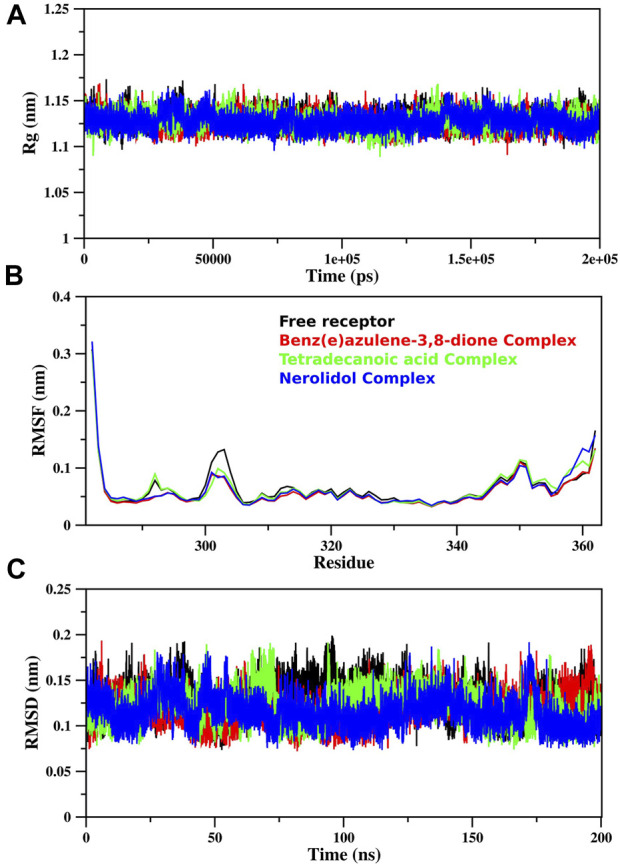
MD simulation of RpfB: The simulation of free RpfB and complexes with three lead molecules after docking analysis using MOE The radius of gyration **(A)**, RMSF **(B)**, and RMSD **(C)** were plotted in the figure. Gromacs scripts were used for the extraction of raw data and plotted using Grace Software.

**FIGURE 8:**
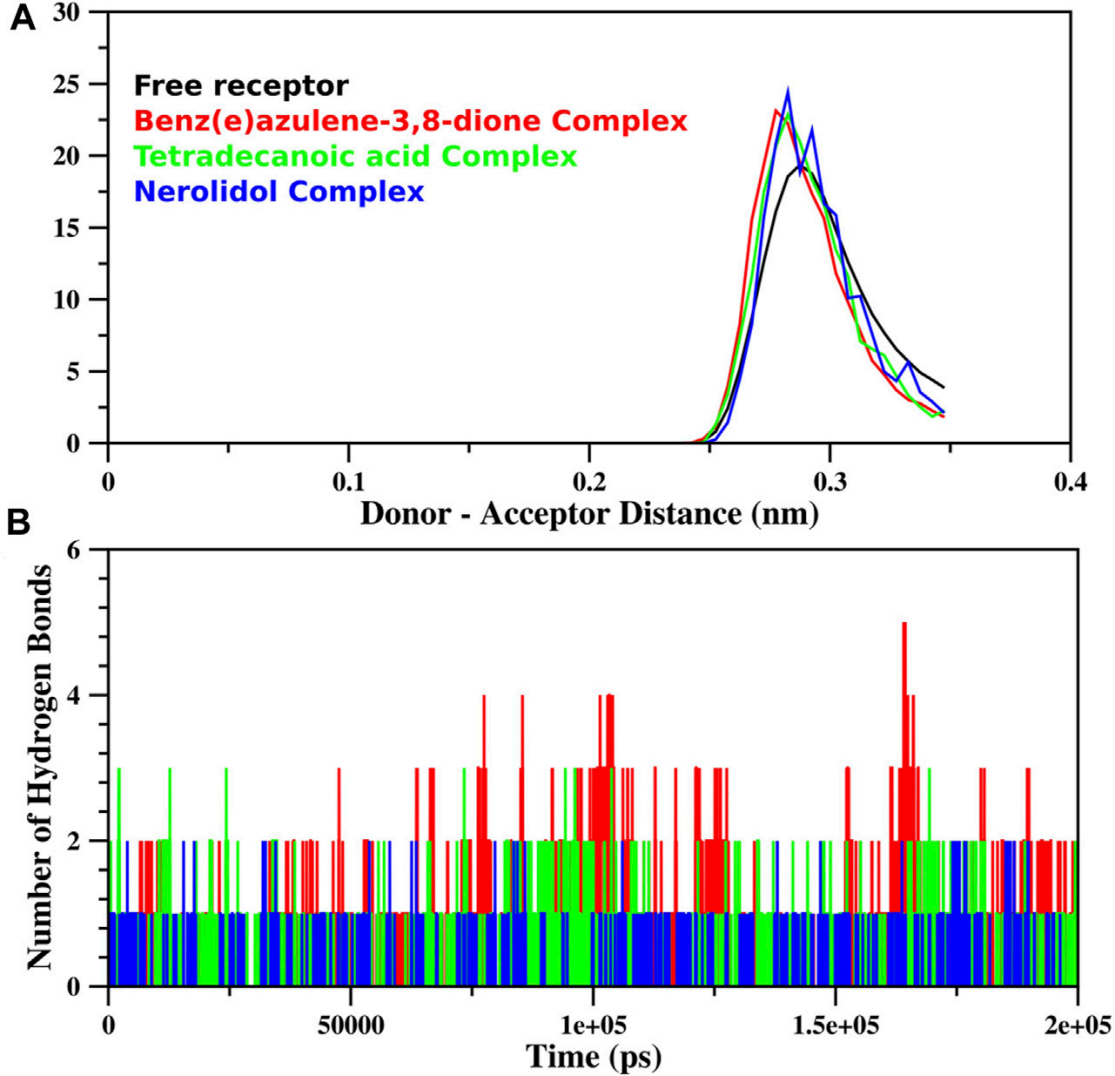
Hydrogen bond analysis of the trajectory obtained during MD simulation of RpfB is shown as a function of distance between **(A)** donor and acceptor (nm) and **(B)** simulation time (ps). The total number of hydrogen bonds between receptors and ligands was specified during gromacs command execution. The color profile represents the respective ligands as indicated in the above plot.

The MD simulation of free proteins as receptors and then complexes with the three best-ranked ligands was subjected to 200 ns to ensure the full set of discrete conformational changes and target-bound and target-unbound conformational states. The radius of gyration (Rg), RMSF, and RMSD analyses as functions of time (ns) and residues, respectively, were analyzed (Figure 7). It is important to mention that monomer assembly was used for simulation. Figure 7A represents the degree of compactness; the free RpfB complexed with Nerolidol, Benz(e)azulene-3,8-dione and Tetradecanoic acid was plotted as a function of simulation time. The formation of complexes slightly increases the compactness of the system, but overall, the same and stable profile was observed during the course of simulation. A similar pattern was also observed for RMSD (Figure 7C), and no significant variations were seen during the entire course of simulation, indicating stable interactions of the ligands with the bacterial protein. Hydrogen bonds play an important role in the binding interaction between the ligand and protein. Hydrogen bonds between the three ligands and the receptor were analyzed and plotted as a function of simulation time (Figure 8). We have observed a prominent change in the H-bond donors and acceptors of the free and complexed protein. All the three selected lead compounds increased the number of H bonds acceptor and donors after binding with the receptor proteins. It can be seen from Figure 8 that Benz(e)azulene-3,8-dione form a high number of H bonds in comparison to the other two ligands. However, it is also pertinent to note that the consistency in H bonds with the simulation time was higher in the case of Nerolidol (Figure 8). The number of H bonds and their behavior during the simulation time further indicated the stability of the ligand’s protein complexes.

**FIGURE 9:**
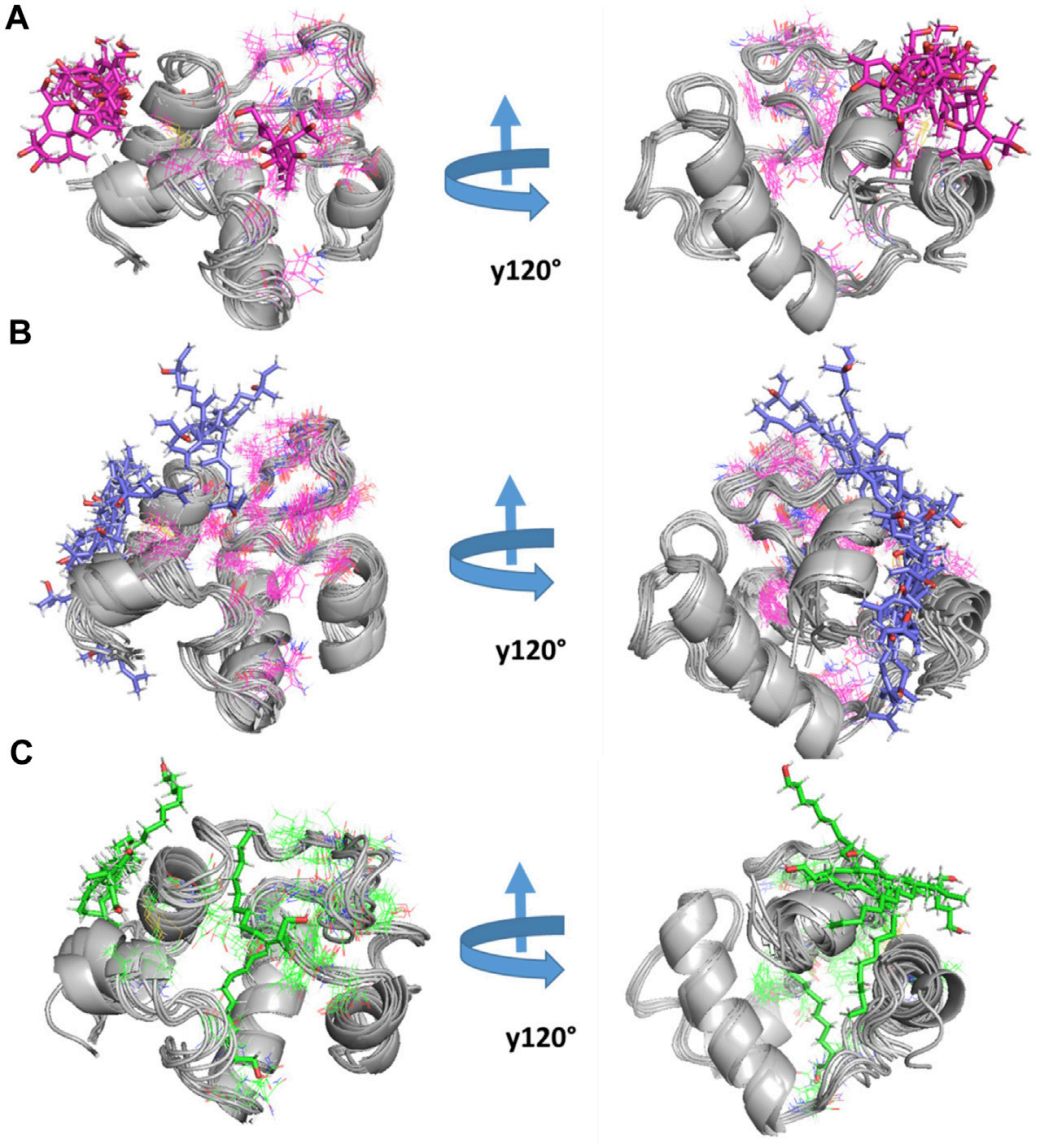
MD simulation of RpfB: The three top-ranked ligands were simulated with RpfB using crystal structures (4EMN) as receptors. The complex was constructed using MOE software. After simulation for 200 ns using Gromacs, snap shots at different time intervals (ns) were presented in the figure. **(A)** Represents Benz, **(B)** Nerolidol, and **(C)** represents Tetradecanoic Acid in complex with the receptor.

The RMSF analysis of the free RpfB and after complexation with ligands shows variation in different regions (Figure 7B). The degree of perturbation decreases for residues E292-N296, W298-G308, and Q313-W316. The region W298-G308 is the binding pocket as defined by literature and docking experiments (Chouhan et al., 2023). However, the other perturbations in the other two regions were difficult to interpret. To have detailed insight and a plausible explanation, the MD trajectory was transformed into an ensemble analysis. All the structures of the ensembles were analyzed after a 10 ns interval. The ensemble analysis of complexes is important for the recognition of target active sites and ligand stability as a function of time. Figure 8 represents data obtained from the MD simulation of RpfB complexed with all other ligands. Figure 9A represents Benz(e)azulene-3,8-dione, (B) Nerolidol and (C) Tetradecanoicacid in complex with the receptor. In the beginning of the stimulation, the ligand stayed bound to the initial site as defined by docking experiments. For example, in Tetradecanoic acid the initial position of the ligand was maintained for 10 ns, and after 20 ns the ligand shifted towards the binding site constituted by C291, I288, Ala287, I284, R358 and A359 and stayed there for the next 60 ns. The ligand Tetradecanoic acid stayed unbound for 80 ns moved around the receptors, and showed transient interaction for a very short period of time with N-terminal residues. The ligand Tetradecanoic acid shifted towards the active site of the C terminal helix at a time interval of 150 ns and stayed bound for the next 10 ns. A similar pattern was found for Benzo and Nerolidol. The ensemble analysis showed that the ligands changing their position between the defined active site and these: C291, I288, Ala287, I284, R358, and A359. These observations may need further analysis to decode the movement of the ligands between these two different sites.

The binding free energies as obtained from alchemical free methods shows preferentially affinity of ligands towards binding site (Table 4). The Nerolidol and Benz(e)azulene-3,8-dione shows comparatively greater free energies then tetradecanoic acid as shown by table. These results shows agreement with the hydrogen bonds tendencies as obtained from MD simulations and comparable scaffold of compounds.

**TABLE 4:** The binding free energies of the top three compounds and receptor complexes.

Sr. No.	Compound name	ΔG (kcal/mol)
1	Nerolidol	−14.93 ± 0.34
2	Tetradecanoic acid	−13.86 ± 0.41
3	Benz(e)azulene-3,8-dione	−15.96 ± 0.63

## Conclusion

It is of utmost importance to comprehend the pathogenesis of TB, which necessitates the revival of Mtb. This is because latent TB affects a staggering one-third of the global population. The resuscitation of Mtb from dormancy is primarily caused by resuscitation-promoting factor (RpfB), making it an attractive target for developing TB drugs in light of latent TB effects. Glu292 of RpfB plays a central role in catalytic activity, and targeting it effectively inhibits RpfB activity, which stops the growth of bacteria in dormancy. This study investigated the phytochemicals from *G. sylvestre* against the RpfB for the discovery of putative novel therapeutic agents for TB. Our results demonstrated that of the reported secondary metabolites from *G. sylvestre*, at least ten compounds showed strong affinity to inhibit RpfB by interacting with the crucial Glu292 residue of the active site. The stability of the ligands with the receptor was further confirmed by the MD simulation at 200 ns, which indicated that the ligands and receptor are highly stable. The top leads were found to follow all the required druggability criteria and possess the least toxicity towards the host. In addition to their direct anti-TB potential, the hits exhibited expanded biological spectra that might further assist in TB treatment. The compounds explored in this study are worthy enough to be subjected to experimental validation. This study also provides a promising for the research on the pharmacological potential of *G. sylvestre.*

## Data Availability

The original contributions presented in the study are included in the article/Supplementary Material, further inquiries can be directed to the corresponding authors.
