# Supplementary material for: Computer-aided identification of Mycobacterium tuberculosis resuscitation-promoting factor B (RpfB) inhibitors from Gymnema sylvestre natural products
Source: Front Pharmacol. 2023 Nov 29;14:1325227. doi: 10.3389/fphar.2023.1325227 (PMC10716330; doi:10.3389/fphar.2023.1325227)
Supplement: Supplementary file 1 [file Table1.DOCX]

**Table S1.** Secondary metabolites of *Gymnema sylvestre* plant used for the preparation of an in-house library.

| **Sr. No.** | **Compound** | **Structure** | **Docking score**  **(s) kcal/mol** | **Ref** |
| --- | --- | --- | --- | --- |
| 1- | Catechol |  | -3.55921388 | [1] |
| 2- | Tetradecanoic acid |  | -5.83323097 | [1] |
| 3- | Bicyclo[1.2.1]heptane, 1,2-dimethyl |  | -6.1394887 | [1] |
| 4- | 6-Octen-1-ol, 3,7-dimethyl-, formate |  | -5.18349934 | [2] |
| 5- | Bicyclo[3.1.1]heptane, 2,6,6-trimethyl- |  | -4.06870031 | [2] |
| 6- | n-Hexadecanoic Acid |  | -5.67417908 | [2] |
| 7- | Isophytol |  | -6.63704586 | [2] |
| 8- | α-Santoline alcohol |  | -4.88792324 | [2] |
| 9- | DL- Ephedrine |  | -4.53232718 | [2] |
| 10- | Squalene |  | -8.14170074 | [2] |
| 11- | δ-Tocopherol |  | -6.88195467 | [2] |
| 12- | Nerolidol |  | -5.9813323 | [2] |
| 13- | Vita min E |  | -6.35299397 | [2] |
| 14- | β-Amyrin |  | -5.1087923 | [2] |
| 15- | Stigmasterol |  | -6.05986786 | [2] |
| 16- | Azulene, 1,2,3,5,6,7,8,8a-octahydro-1,4-dimethyl-7-(1-methylethenyl |  | -4.53128719 | [2] |
| 17- | Hop -22(29)-en-3β-ol |  |  | [2] |
| 18- | Cedrene-V6 |  | -4.58174038 | [2] |
| 19- | Propenamide |  | -3.27390742 | [3] |
| 20- | Diethyl Phthalate |  | -4.96039629 | [3] |
| 21- | 1,3 Dioxalane |  | -3.26720452 | [1] |
| 22- | Thiazole |  | -3.15979528 | [4] |
| 23- | Dimethy 2-methoxy hexane |  |  | [4] |
| 24- | Methyl cyclohexane propionate |  | -4.70301437 | [4] |
| 25- | Tetratriacontane |  | -7.71303463 | [4] |
| 26- | Phthalic Acid |  | -4.25505495 | [5] |
| 27- | pentanoic Acid |  | -3.57116866 | [5] |
| 28- | Etodolac Methyl Ester |  | -5.41358614 | [6] |
| 29- | Oleic Acid |  | -6.28390408 | [1] |
| 30- | Eicosane |  | -6.59507275 | [1] |
| 31- | Methyl tetra decanoate |  | -6.05920172 | [1] |
| 32- | Azabicyclo octanol |  | -4.01363468 | [1] |
| 33- | 1,2 Benzene dicarboxylic acid |  | -4.35797119 | [1] |
| 34- | Hexacosane |  | -7.07823277 | [1] |
| 35- | Ergosterol |  | -6.3261919 | [2] |
| 36- | Cholestan 3 one |  | -6.00551367 | ][7] |
| 37- | Benzoquinoline |  | -4.29197311 | [5] |
| 38- | Benzazirene Carboxylic Acid |  |  | [3] |
| 39- | Octadecane |  | -6.28959608 | [3] |
| 40- | 1,5-Naphthyridin-4-amine |  | -3.70975113 | [3] |
| 41- | Triacontane |  | -8.13893223 | [3] |
| 42- | 1 Methyl Cyclopentanol |  | -5.71355391 | [3] |
| 43-- | Taraxasterol |  | -5.15691519 | [2] |
| 44- | 1,8-Cineole |  | -3.94441938 | [6] |
| 45- | Octanol |  | -4.64584351 | [6] |
| 46- | Beta Elemene |  | -5.22981071 | [7] |
| 47- | Dodecanol |  | -6.01317739 | [7] |
| 48- | Methyl Eugenol |  | -4.42589474 | [3] |
| 49-- | 2-Pentadecanone |  | -6.04765749 | [3] |
| 50- | 4-Ethyl Guaiacol |  | -4.3121047 | [3] |
| 51- | 8-Dodecenol |  | -5.16775513 | [2] |
| 52- | Hexahydrofarnesyl |  | -6.3225441 | [4] |
| 53- | Acetone |  | -2.99377918 | [4] |
| 54- | Eugenol |  | -4.32901907 | [4] |
| 55- | 4 Ethyl Phenol |  | -3.79724216 | [5] |
| 56- | Tetradecanol |  | -5.27130127 | [6] |
| 57- | 4 vinyl Guaiacol |  | -4.08308792 | [1] |
| 58- | Methyl Palmitate |  | -6.46786213 | [1] |
| 59- | Ethyl Palmitate |  | -6.34529066 | [1] |
| 60- | 1 Pentadecanol |  | -5.96635532 | [1] |
| 61- | Tetradecadiene-2,13 |  | -5.04740429 | [1] |
| 62- | 9,12,15-Octadecatrienal |  | -6.35638142 | [2] |
| 63- | 3-Methoxyacetophenone |  | -3.75238848 | [2] |
| 64- | 2,3,5,6-Tetrafluoroanisole |  | -3.67932916 | [2] |
| 65- | 1,2,3,4-Cyclohexanetetrol |  | -3.91581321 | [2] |
| 66- | A'-Neogammacer-22(29)-ene |  | -5.1051383 | [2] |
| 67- | Cholane-5,20(22)-diene-3b-phenoxy |  | -6.29461813 | [2] |
| 68- | Spiro[cyclopropane-1,2'-[6.7]diazabicyclo[3.2.2]non-6-ene] |  | -3.93520141 | [2] |
| 69- | Cycloheptane, 4-methylene-1-methyl-2-(2-methyl-1-propen-1-yl)-1-  vinyl- |  | -4.66343737 | [2] |
| 70- | d-quercitol, |  | -4.08129835 | [4] |
| 71- | Anthraquinones |  | -4.18649435 | [4] |
| 72- | tartaric acid |  | -4.22646713 | [4] |
| 73- | formic acid |  | -2.88528752 | [4] |
| 74- | butyric acid, |  | -3.60978079 | [4] |
| 75- | Calcium oxalate |  |  | [4] |
| 76- | Gymnemanol |  |  | [4] |
| 77- | hydroxycinnamic acid |  | -4.86286736 | [5] |
| 78- | Alpha Tocopherol Acetate |  | -8.26282597 | [1] |
| 79- | Hydroquinone |  | -3.65805769 | [5] |
| 80- | A-Norcholestan-3-one, 5-ethenyl |  | -6.58468485 | [2] |
| 81- | Gymnemic acid VIII |  | -7.12902546 | [7] |
| 82- | Gymnemic acid VI |  | -6.75290728 | [3] |
| 83- | Gymnestrogenin |  |  | [7] |
| 84- | Lupeol |  |  | [4] |
| 85- | Parabin |  | -4.10629702 | [6] |
| 86- | Quercitol |  | -4.82462978 | [4] |
| 87- | Conduritol A |  | -3.91844058 | [4] |
| 88- | Gymnemoside A |  | -6.72475386 | [8] |
| 89- | Gymnemoside B |  | -6.68001509 | [3] |
| 90- | Gymnemoside C |  |  | [3] |
| 91- | Gymnemoside D |  |  | [3] |
| 92- | Gymnemoside E |  | -8.08817959 | [3] |
| 93- | Gymnemoside F |  | -8.25280094 | [3] |
| 94- | 3 Ethenyl-3,6,6.7-tetramethyl-2,3a,4,5-tetrahydro-1H-Indene |  | -4.57530451 | [6] |
| 95- | Phytyl Acetate |  | -6.77823544 | [6] |
| 96- | Gamma Tocopherol |  | -6.80919027 | [6] |
| 97- | Hopeaphenol |  | -6.14120722 | [1] |
| 98- | 1,6,10,14,18,22-Tetracosahexaen-3-ol, 2,6,10,15,19,23-hexamethyl-, (all-E)- |  | -8.16238022 | [2] |
| 99- | Cis-Nerolidol |  | -5.45901108 | [2] |
| 100- | Diheptan-3-yl Benzene 1,2 Dicarboxylate |  | -7.30432844 | [6] |
| 101- | 2-Palmitoglycerol |  | -6.2947526 | [2] |
| 102- | Santolina Alcohol |  | -4.45178318 | [2] |
| 103- | Bicyclo[3.1.1]heptane, 2,6,6-trimethyl |  | -5.49569035 | [2] |
| 104- | Bicyclo[2.2.1]heptane, 1,3,3-trimethyl |  | -4.92055559 | [2] |
| 105- | 1,2,3,4-Cyclohexanetetrol |  | -3.91242266 | [6] |
| 106- | 2,3,5,6-Tetrafluoroanisole |  | -3.68285751 | [6] |
| 107- | 8,11,14 Ecosatrienoic acid |  | -6.70273685 | [6] |
| 108- | Methyl Linolenate |  | -6.51184559 | [6] |
| 109- | Acetophenone |  | -3.86727166 | [6] |
| 110- | Pentacosane |  | -6.68917227 | [6] |
| 111- | Propane, 1,1-diethoxy-3-iodo |  | -4.76046085 | [6] |
| 112- | Methyl cis cis 11,14-Eicosadienoate |  | -6.91906118 | [6] |
| 113- | Methyl salicylate |  | -4.2574296 | [2] |
| 114- | Ethyl Octadec-9-enoate |  | -6.67737532 | [2] |
| 115- | Indole |  | -4.0991168 | [8] |
| 116- | Germacrene A |  | -4.72679329 | [8] |
| 117- | Gymnemic Acid I |  | -6.76740503 | [7] |
| 118- | Gymnemic Acid II |  | -5.64104986 | [7] |
| 119- | Gymnemic Acid III |  | -6.05563831 | [7] |
| 120- | Gymnemic Acid 4 |  | -5.95438528 | [7] |
| 121- | Gymnemasin A |  | -6.65773582 | [6] |
| 122- | Gymnemasin B |  | -7.71715832 | [6] |
| 123- | Gymnemasin C |  | -5.99085093 | [6] |
| 124- | Gymnemasin D |  | -6.85996056 | [8] |
| 125- | Gymnemic acid V |  | -F369508991 | [1] |
| 126- | Gymnemic acid X |  | -6.86802149 | [1] |
| 127- | Gymnemic acid XII |  | -6.51239872 | [9] |
| 128- | Gymnemic acid VII |  |  | [9] |
| 129- | Methylene Cyclohexane |  | -3.6308465 | [9] |
| 130- | 1-OH-1,7-dimethyl-4-isopropyl-2,7-cyclodecadiene |  | -4.65116119 | [9] |
| 131- | Benz(e)azulene-3,8-dione |  | -5.35518837 | [4] |
