# Supplementary figures and images for: Computer-aided identification of Mycobacterium tuberculosis resuscitation-promoting factor B (RpfB) inhibitors from Gymnema sylvestre natural products
Source: Front Pharmacol. 2023 Nov 29;14:1325227. doi: 10.3389/fphar.2023.1325227 (PMC10716330; doi:10.3389/fphar.2023.1325227)

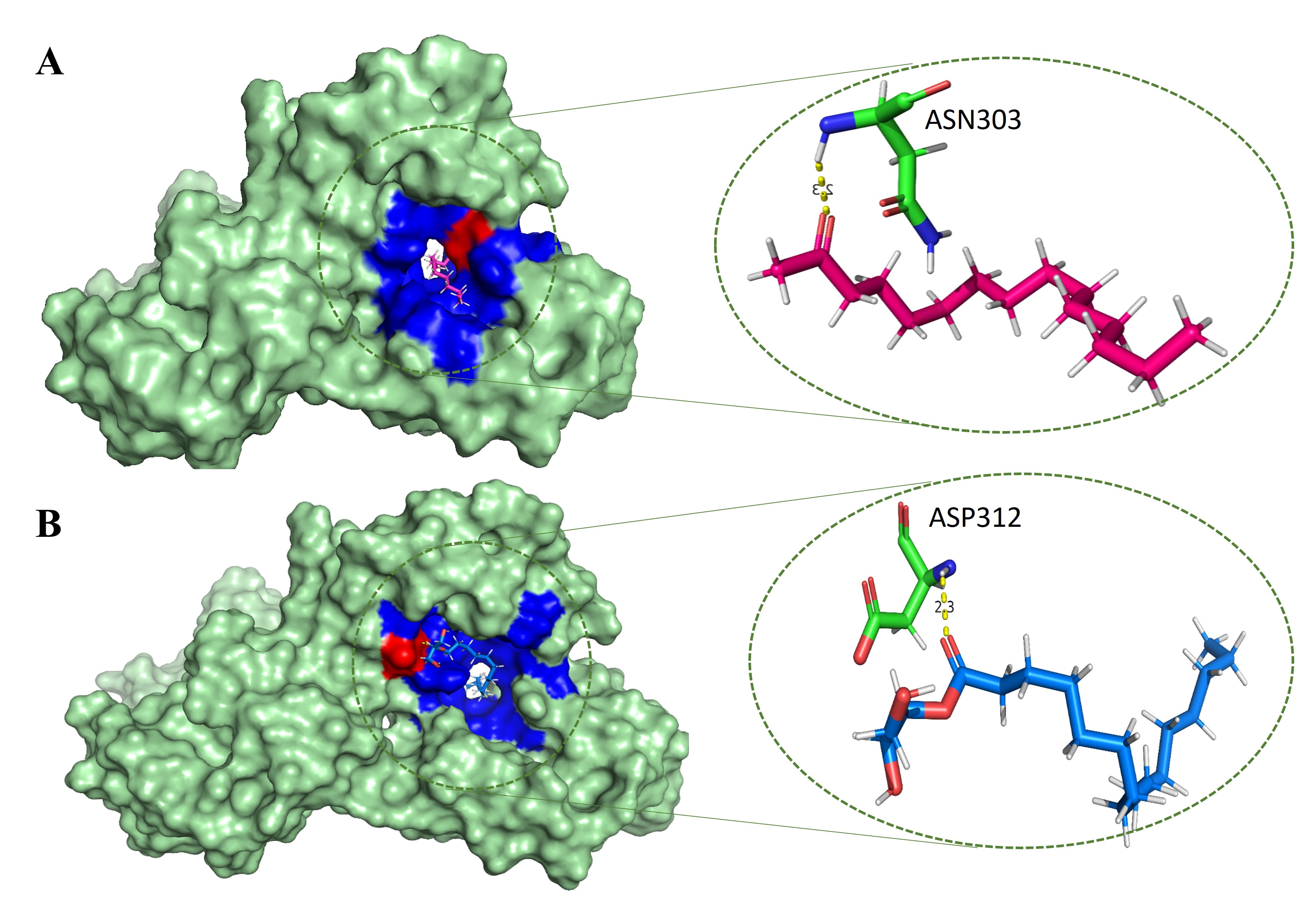

Supplement: Supplementary file 2 [file Image1.JPEG]

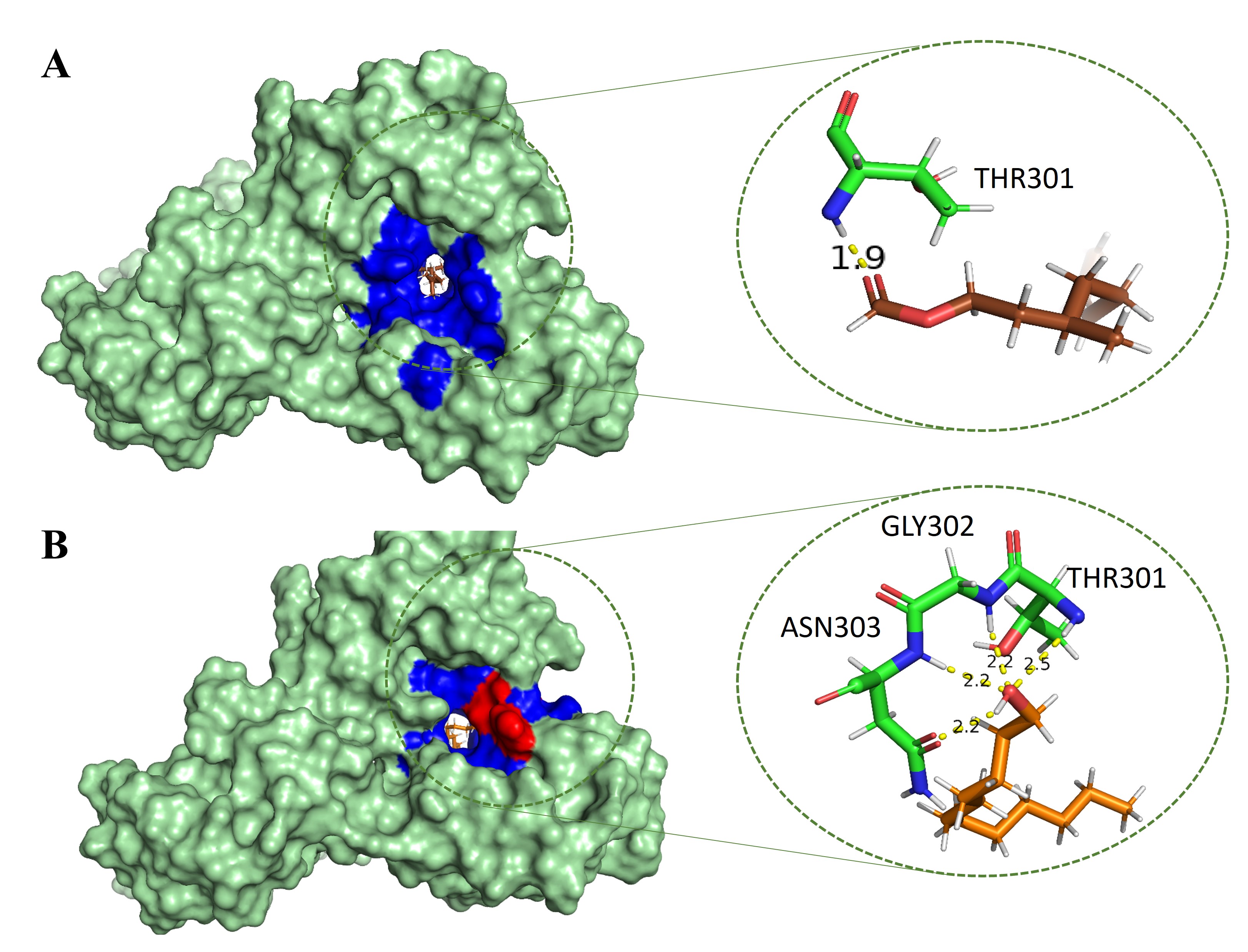

Supplement: Supplementary file 3 [file Image2.JPEG]
