# Supplementary material for: Computer-aided identification of Mycobacterium tuberculosis resuscitation-promoting factor B (RpfB) inhibitors from Gymnema sylvestre natural products
Source: Front Pharmacol. 2023 Nov 29;14:1325227. doi: 10.3389/fphar.2023.1325227 (PMC10716330; doi:10.3389/fphar.2023.1325227)
Supplement: Supplementary file 4 [file Table2.DOCX]

**Table S2.** Molecular docking and interaction analysis of top lead compounds.

| **Sr. No.** | **Compound**  **Name** | **Docking score** (**kcal/mol)** | **Hydrogen bond** | **Hydrophobic interaction** |
| --- | --- | --- | --- | --- |
|  | 2-Palmitoglycerol | -6.294 | Asp312 | Glu292, Asn303, Gly304, gln310, Ala293, Tyr303, Phe 311, Thr315, |
|  | Benz(e)azulene-3,8-dione | -5.355 | Glu292 | Cys291, ALA293, Gly294, Asn296, Ile299, TYR305, GLN310, ASP312, TRP352, PRO353, CYS355, ASN301, |
|  | Nerolidol | -5.981 | Glu292 | Cys291, Ala293, Gly294, Ile299, Asn303, Tyr305, Gln310, Trp287, Cys355 |
|  | Dodecanol | -6.013 | Tyr305,  Asn303,  Gly302 | Glu292, ALA293, ILE299, ASN300, GLY304, TYR306, GLN310, CYS291, ASN303 |
|  | Tetradecanoic acid | -5.833 | Gln310 | CYS291, GLU292, ALA293, GLY294, ILE299, THR301, ASN303, TYR305, VAL309, GLN310, PRO353, VAL354, CYS355 |
|  | 6-Octen-1-ol-3,7-dimethylformate | -5.183 | Thr301 | Cys291, Glu292, Ala293, Gly294, Asn296, Ile299, Asn303, Tyr305, Gln310, Gly350, Val354, Gly302, Tyr306 |
|  | 8-Dodecenol | -5.167 | Glu292 | Cys291, Ala292, Gly294, Ile299, Asn303, Tyr305, Gln310, |
|  | Methyltetradecanoate | -6.059 | Gln310 | CYS291, ALA293, GLY294, ILE294, ASN303, TYR305, GLN310, GLY350, ALA351, PRO353 |
|  | Tetradecenol | -5.271 | Glu292 | CYS291, ALA292, ILE294, TYR305, GLN310, GLY302, ALA350, CYS355 |
|  | 2-pentadecanone | -6.047 | Asn303 | CYS291, GLU292, ALA293, ILE299, ASN300, THR301, GLY302, GLY304, TYR305, TYR306, GLY307, GLN310, GLY350, ALA350, CYS355, ASN303 |
