# Supplementary material for: Computer-aided identification of Mycobacterium tuberculosis resuscitation-promoting factor B (RpfB) inhibitors from Gymnema sylvestre natural products
Source: Front Pharmacol. 2023 Nov 29;14:1325227. doi: 10.3389/fphar.2023.1325227 (PMC10716330; doi:10.3389/fphar.2023.1325227)
Supplement: Supplementary file 5 [file Table3.DOCX]

**Table S3.** Toxicity analysis of top compounds by Protox 2 server.

| **Sr. No.** | **Compound**  **Name** | **LD50 value**  **(mg/kg)** | **Cytotoxicity**  **probability** | **Hepatotoxicity** |
| --- | --- | --- | --- | --- |
| 1 | Nerolidol | 5000 | 0.81 | 0.81 |
| 2 | Dodecanol | 1000 | 0.83 | 0.90 |
| 3 | Benz(e)azulene-3,8-dione | 50 | 0.74 | 0.81 |
| 4 | 2-pentadecanone | 5000 | 0.73 | 0.69 |
| 5 | 2-Palmitoglycerol | 5000 | 0.86 | 0.91 |
| 6 | Tetradecanoic acid | 900 | 0.74 | 0.52 |
| 7 | 6-Octen-1-ol-3,7-dimethylformate | 8400 | 0.82 | 0.71 |
| 8 | 8-Dodecenol | 5600 | 0.81 | 0.87 |
| 9 | Methyltetradecanoate | 5000 | 0.73 | 0.58 |
| 10 | Tetradecenol | 2070 | 0.86 | 0.84 |
| **Standard** | **Benzamidine** | 930 | 0.76 | 0.67 |
