# Supplementary material for: Computer-aided identification of Mycobacterium tuberculosis resuscitation-promoting factor B (RpfB) inhibitors from Gymnema sylvestre natural products
Source: Front Pharmacol. 2023 Nov 29;14:1325227. doi: 10.3389/fphar.2023.1325227 (PMC10716330; doi:10.3389/fphar.2023.1325227)
Supplement: Supplementary file 6 [file Table4.DOCX]

**Table S4.** Pass Prediction Analysis of the top hits.

|  | **Anti-eczematic** | **Anti-seborrheic** | **Anti-hypoxic** | **Anti-mutagenic** | **Anti-**  **inflammatory** | **Anti-**  **secretoric** | **Anti-**  **ulcerative** |
| --- | --- | --- | --- | --- | --- | --- | --- |
| Tetradecanoic acid | + | + | + | + | + | + | - |
| 6-Octen-1-ol, 3,7-dimethylformate | - | - | - | - | - | - | + |
| Nerolidol | + | - | - | - | + | + | + |
| Methyltetradecanoate | + | + | - | - | + | - | - |
| Dodecanol | + | + | + | - | - | - | - |
| 2-Pentadecanone | + | + | - | - | - | - | - |
| 8-Dodecenol | + | + | + | + | - | + | - |
| Tetradecenol | + | + | + | - | - | - | - |
| 2-Palmitoglycerol | + | + | + | - | - | + | - |
| Benz(e)azulene-3,8-dione | - | - | - | - | - | - | - |

Plus (+) and negative (-) signs indicate the presence and absence of the activity in the compounds, respectively.
